# Asthmatic Bronchial Matrices Determine the Gene Expression and Behavior of Smooth Muscle Cells in a 3D Culture Model

**DOI:** 10.3389/falgy.2021.762026

**Published:** 2021-11-26

**Authors:** Selma Ben Hamouda, Maria Angélica Miglino, Gustavo de Sá Schiavo Matias, Guy Beauchamp, Jean-Pierre Lavoie

**Affiliations:** ^1^Faculty of Veterinary Medicine, Université de Montréal, Quebec City, QC, Canada; ^2^School of Veterinary Medicine and Animal Sciences, University of São Paulo, São Paulo, Brazil

**Keywords:** asthma, 3D culture, extracellular matrix, airway smooth muscle, recellularization, phenotype

## Abstract

Asthma is associated with increased deposition and altered phenotype of airway smooth muscle (ASM) cells. However, little is known about the processes responsible for these changes. It has been suggested that alterations of the extracellular matrix (ECM) contribute to the remodeling of ASM cells in asthma. Three-dimensional matrices allow the *in vitro* study of complex cellular responses to different stimuli in a close-to-natural environment. Thus, we investigated the ultrastructural and genic variations of ASM cells cultured on acellular asthmatic and control bronchial matrices. We studied horses, as they spontaneously develop a human asthma-like condition (heaves) with similarities to chronic pulmonary changes observed in human asthma. Primary bronchial ASM cells from asthmatic (*n* = 3) and control (*n* = 3) horses were cultured on decellularized bronchi from control (*n* = 3) and asthmatic (*n* = 3) horses. Each cell lineage was used to recellularize six different bronchi for 41 days. Histomorphometry on HEPS-stained-recellularized matrices revealed an increased ASM cell number in the control cell/control matrix (*p* = 0.02) and asthmatic cell/control matrix group (*p* = 0.04) compared with the asthmatic cell/asthmatic matrix group. Scan electron microscopy revealed a cell invasion of the ECM. While ASM cells showed high adhesion and proliferation processes on the control ECM, the presence of senescent cells and cellular debris in the asthmatic ECM with control or asthmatic ASM cells suggested cell death. When comparing asthmatic with control cell/matrix combinations by targeted next generation sequencing, only *AGC1* (*p* = 0.04), *MYO10* (*p* = 0.009), *JAM3* (*p* = 0.02), and *TAGLN* (*p* = 0.001) were differentially expressed out of a 70-gene pool previously associated with smooth muscle remodeling. To our knowledge, this is the first attempt to evaluate the effects of asthmatic ECM on an ASM cell phenotype using a biological bronchial matrix. Our results indicate that bronchial ECM health status contributes to ASM cell gene expression and, possibly, its survival.

## Introduction

The asthmatic airways undergo remodeling including loss of epithelial cell integrity, mucus gland and goblet cell hypertrophy, extracellular matrix (ECM) fibrosis, angiogenesis, and increased airway smooth muscle (ASM) mass (1, 2). ASM enlargement was recognized as the main feature of airway wall thickness in asthma as early as 1922 (3). Later studies determined its pivotal role in asthmatic bronchial remodeling and hyperreactivity (4, 5). However, the source of ASM remodeling remains debated and includes epithelial to mesenchymal transition (6, 7), reduced cell apoptosis, and ASM cells hypertrophy and hyperplasia (8–12).

Tissue engineering was developed to create substitutes for injured tissues or organs (13, 14). Cells cultured on 3D substrates have a different behavior than those from 2D cultures and better mimic the *in vivo* environment (15–17). Therefore, protocols using 3D substrates have been adapted for studies on cellular phenotypes in disease and in response to drugs (18–20). It has been reported that compared to 2D cell culture, human umbilical artery smooth muscle cells cultured in hanging drops have an altered gene expression pattern and reduced protein levels of PCNA and increased levels of α-SMA when compared to 2D standard cell culture (21). Also, 3D co-cultures influence the phenotype of smooth muscle cells. For instance, 3D co-culturing vascular smooth muscle cells and endothelial cells, the vascular smooth muscle cells went from quiescence to contractile phenotype (22). Therefore, the composition of the bronchial scaffold and its organization could affect ASM cell development and behavior (23).

Factors triggering ASM remodeling and hyperresponsiveness remain understudied and, to the best of our knowledge, have never been explored under 3D culture conditions (24–26). In this study, we aimed to investigate the influence of bronchial extracellular matrix on the gene expression and phenotype of asthmatic ASM using a 3D cell culture. We studied an equine bronchial scaffold using decellularization and recellularization protocols previously developed (27) because of similarities reported between human and equine asthma (28, 29).

## Methods

A diagram summarizing the experimental design is reported in Supplementary Material 1.

### Animals

Archived lung tissues and airway smooth muscle cells from *three* asthmatic and *three* healthy control horses, aged 10–12 years, from a tissue bank (htttps://www.btre.ca) were studied. An ante-mortem diagnosis of severe asthma was based on history of periods of labored breathing at rest, lung function results (pulmonary resistance R_L_ ≥ 1 cm H_2_O/L/s), and bronchoalveolar lavage fluid (BALF) cytology (neutrophils >10%). Control horses had no history of lung diseases, and had normal lung function and BALF cytology (30) (Supplementary Material 2). The experimental protocol was approved by the ethical committee of the University of Montreal, with number Rech-1578.

### Airway Smooth Muscle Cells: Isolation and Culture

Airway smooth muscle cells were isolated and cultured in the 1st h after collection from bronchial first carina as previously described (27). Briefly, ASM was digested in Dulbecco's Modified Eagle Medium (DMEM)/F12 Nutrient Mix Medium (Thermo Fisher Scientific, Waltham, MA, United States) supplemented with 0.125 U/ml Collagenase H (Sigma Aldrich, St. Louis, MO, United States), 1 mg/ml trypsin inhibitor (Sigma Aldrich, St. Louis, MO, United States), 1 U/ml elastase (Worthington Biochemical, Lakewood, NJ, United States), in the presence of penicillin-streptomycin (Wisent Inc., Saint-Jean-Baptiste, QC, Canada) and Fungizone (Thermo Fisher Scientific, Hampton, NH, United States) for 3 h under rotational movement in a humidified environment at 37°C with 5% CO_2_. The cells were then seeded in ventilated cell culture flasks and cultured in a DMEM/F-12 medium complemented with fetal bovine serum (FBS) at 37°C, and 5% CO_2_ upon passage 2 (P2). The cells were then frozen in a solution of FBS (Wisent Inc., Saint-Jean-Baptiste, QC, Canada) and 1% DMSO (Thermo Fisher Scientific, Hampton, NH, United States), and kept in liquid nitrogen until needed. The cell vials were quickly unfrozen in a 37°C bath, seeded in ventilated cell culture flasks, and cultured in a DMEM/F-12 medium and passed until passages 4 to 5 (P4–P5).

### Fibroblasts: Isolation and Culture

Fibroblasts isolated from the first bronchial carina and the endoderm of the brachial triceps of control horses were cultured to evaluate the effects of a possible contamination of ASM cells by fibroblasts. In brief, within 90 min after collection, the cells were digested in DMEM complemented with 0.2 U/ml Collagenase H and 10 mM CaCl_2_ under the environmental conditions for ASM cells described above. The fibroblasts were then seeded in ventilated cell culture flasks and cultured in DMEM complemented with 10% heat-inactivated FBS at 37°C and 5% CO_2_ until passage 2. The cells were then frozen in a solution of FBS (Wisent Inc., Saint-Jean-Baptiste, QC, Canada) and 1% DMSO (Thermo Fisher Scientific, Hampton, NH, United States) and kept in liquid nitrogen until needed. Primary equine fetal fibroblasts courtesy of Dr. Lawrence Smith were also studied. Unfrozen cells were then cultured in DMEM and passed until passages P4–P5.

### Decellularization and Recellularization of the Bronchi

The protocol used to decellularize and recellularize the bronchi has been reported previously (27). In brief, bronchi from second to fourth generation were manually dissected from the surrounding lung tissues within 2 h after euthanasia of the horses. The bronchi were then snap-frozen in liquid nitrogen and kept at −80°C until they were used. The bronchi were thawed in sterile PBS 1X at room temperature, decellularized and recellularized using protocols described previously. Briefly, two consecutive cycles of a detergent (Triton 1X, sodium deoxycholate, and sodium chloride), and enzymatic (DNase) treatments were followed by sterilization with paracetic acid-ethanol under continued agitation. This protocol was shown to remove all cell material, to maintain the general architecture and protein content on histological staining, to have double stranded DNA <200 bp and of a concentration <50 ng/mg of tissue as recommended (31, 32). Decellularized bronchi were then cut into pieces with a maximum size of 1 × 1 cm and rinsed in sterile PBS 1X. Tissues were then placed on a 24-well plate (Costar, Washington, DC) and seeded with ASM cells or fibroblasts at passages 4 and 5 at a concentration of 158,000 cells/cm^2^ corresponding to 3-fold cell confluence rate on the plate. Each ASM cell lineage (*n* = 6) and fibroblasts (*n* = 5) was used to recellularize four replicates of each tissue (*n* = 6) (Table 1).

**Table 1:** Decellularized biologic bronchial matrices from control (*n* = 3) and asthmatic horses (*n* = 3) were recellularized with primary bronchial airway smooth muscle (ASM) cells from the same control (*n* = 3) and asthmatic horses (*n* = 3).

**Control matrices/** **control ASM cells**	***n***	**Control matrices/** **asthmatic ASM cells**	***n***	**Asthmatic matrices/** **control ASM cells**	***n***	**Asthmatic matrices/** **asthmatic ASM cells**	***n***
Horse 1 matrix/ Horse 1 cells	4	Horse 1 matrix/ Horse 4 cells	4	Horse 4 matrix/ Horse 1 cells	4	Horse 4 matrix/ Horse 4 cells	4
Horse 1 matrix/ Horse 2 cells	4	Horse 1 matrix/ Horse 5 cells	4	Horse 4 matrix/ Horse 2 cells	4	Horse 4 matrix/ Horse 5 cells	4
Horse 1 matrix/ Horse 3 cells	4	Horse 1 matrix/ Horse 6 cells	4	Horse 4 matrix/ Horse 3 cells	4	Horse 4 matrix/ Horse 6 cells	4
Horse 2 matrix/ Horse 1 cells	4	Horse 2 matrix/ Horse 4 cells	4	Horse 5 matrix/ Horse 1 cells	4	Horse 5 matrix/ Horse 4 cells	4
Horse 2 matrix/ Horse 2 cells	4	Horse 2 matrix/ Horse 5 cells	4	Horse 5 matrix/ Horse 2 cells	4	Horse 5 matrix/ Horse 5 cells	4
Horse 2 matrix/ Horse 3 cells	4	Horse 2 matrix/ Horse 6 cells	4	Horse 5 matrix/ Horse 3 cells	4	Horse 5 matrix/ Horse 6 cells	4
Horse 3 matrix/ Horse 1 cells	4	Horse 3 matrix/ Horse 4 cells	4	Horse 6 matrix/ Horse 1 cells	4	Horse 6 matrix/ Horse 4 cells	4
Horse 3 matrix/ Horse 2 cells	4	Horse 3 matrix/ Horse 5 cells	4	Horse 6 matrix/ Horse 2 cells	4	Horse 6 matrix/ Horse 5 cells	4
Horse 3 matrix/ Horse 3 cells	4	Horse 3 matrix/ Horse 6 cells	4	Horse 6 matrix/ Horse 3 cells	4	Horse 6 matrix/ Horse 6 cells	4

*Four replicates were produced for each recellularization combination. Horses 1, 2, and 3 are controls and 4, 5, 6 are asthmatic. n: number of replicates*.

The tissues were maintained in culture until day 31 and then transferred to a 6-well plate (Celltreat, Pepperell, MA, United States) until day 41.

### Flow Cytometry: ASM Cell and Fibroblast Evaluation

The ASM cells and fibroblasts were characterized by flow cytometry before and at 31 days of recellularization (transfer day), as described previously (27). Briefly, the cells were stained for intracellular proteins with anti–α-SMA (mouse IgG2a, 1/250; Sigma Aldrich, St. Louis, MO, United States) and anti-desmin (rabbit polyclonal IgG, 1/200; Abcam, Cambridge, United Kingdom,) antibodies for 1 h. The cells were then washed three times and incubated for 30 min in the dark with fluorescent dye–conjugated anti-IgG antibodies. Isotype-matched control antibodies (mouse IgG2a and rabbit IgG) were used as negative control. All signals greater than those of the isotype-matched control antibodies were considered positive, and the degree of staining was evaluated as the mean fluorescence intensity and mean percentage of positive cells for both α-SMA and desmin.

### Histological Assessment of Recellularization (Movat Pentachrome) and Histomorphometry (HEPS)

Two replicates from each tissue/cell combination were studied. The tissues were fixed in 10% formalin, paraffin-embedded, and then sliced at 4.5 μm thickness and stained using the Movat Pentachrome histologic staining protocol as described previously (27). The qualitative assessment of recellularization was based on a visual examination of recellularized tissue sections under an optical microscope at 100 and 200 magnifications using the Panoptiq software (version 2) connected to a Prosilica GT camera (model: GT1920C) mounted on a Leica DM4000 B microscope.

Slides for histomorphometry were stained using the hematoxylin-eosin-phloxine-safran (HEPS) histologic protocol. The slides were scanned at 200 magnification, and a histomorphometry analysis for total cell number was performed blindly using the ImageJ software (Version 1.35c). For each slide, total cell number was determined using the point counting technic and then corrected to the perimeter of the tissue. A mean value was calculated for each two replicates.

### Scan Electron Microscopy of Recellularized Scaffolds

One replicate of each tissue/cell combination was used. Recellularized bronchi were prepared as described previously (27). Briefly, tissues were fixed in 2.5% glutaraldehyde, washed and post-fixed in a 1% aqueous osmium tetroxide solution, and dehydrated in alcohol. The samples were blindly analyzed on LEO 435VP Scanning Electron Microscope at the Advanced Diagnostic Center by Image - CADI - Faculty of Veterinary Medicine and Animal Science, University of São Paulo.

### Targeted Next Generation Sequencing (NGS) for Recellularized Bronchi

One replicate of each tissue/cell combination was used for Ampliseq transcriptome sequencing. Three tissue/cell combinations were used to create a total of 12 libraries that were divided into four groups (control/control, control/asthma, asthma/control, and asthma/asthma), see Table 2 for details. The libraries were constructed using Ion AmpliSeq Library Kit Plus (Life Technologies, Carlsbad, CA, United States) according to the instructions of the manufacturer using a customized panel of 70 equine-specific probes for genes related to asthma remodeling (Supplementary Material 3). Sequencing was performed using Ion Torrent System (Life Technologies, Carlsbad, CA, United States) at Virology Laboratory, Department of Pathology and Microbiology, Faculty of Veterinary Medicine, University of Montreal.

**Table 2:** Composition of the Ampliseq libraries.

**Control matrices/control cells**		**Control matrices/asthmatic cells**		**Asthmatic matrices/control cells**		**Asthmatic matrices/asthmatic cells**	
C1 matrix/C1 cells	Library 1	C1 matrix/A1 cells	Library 4	A1 matrix/C1 cells	Library 7	A1 matrix/A1 cells	Library 10
C1 matrix/C2 cells		C1 matrix/A2 cells		A1 matrix/C2 cells		A1 matrix/A2 cells	
C1 matrix/C3 cells		C1 matrix/A3 cells		A1 matrix/C3 cells		A1 matrix/A3 cells	
C2 matrix/C1 cells	Library 2	C2 matrix/A1 cells	Library 5	A2 matrix/C1 cells	Library 8	A2 matrix/A1 cells	Library 11
C2 matrix/C2 cells		C2 matrix/A2 cells		A2 matrix/C2 cells		A2 matrix/A2 cells	
C2 matrix/C3 cells		C2 matrix/A3 cells		A2 matrix/C3 cells		A2 matrix/A3 cells	
C3 matrix/C1 cells	Library 3	C3 matrix/A1 cells	Library 6	A3 matrix/C1 cells	Library 9	A3 matrix/A1 cells	Library 12
C3 matrix/C2 cells		C3 matrix/A2 cells		A3 matrix/C2 cells		A3 matrix/A2 cells	
C3 matrix/C3 cells		C3 matrix/A3 cells		A3 matrix/C3 cells		A3 matrix/A3 cells	

*Each group contains three libraries as replicates. C, control; A, asthmatic*.

### Statistical Analyses

The values are expressed as mean ± standard error of the means. Results were analyzed using a mixed linear model (SAS v9.4.) for flow cytometry analysis comparing ASM cells before and during recellularization. For histomorphometry analysis, an unpaired *t*-test was performed to compare the asthmatic/asthmatic group with the control/control group, and a paired *t*-test was performed for the other group comparisons. Flow cytometry results and next generation sequencing analysis were compared with unpaired *t*-tests (GraphPad Prism 8.4.3). Values of *P* ≤ 0.05 were considered significant.

## Results

### Flow Cytometry

**Figure 1:**
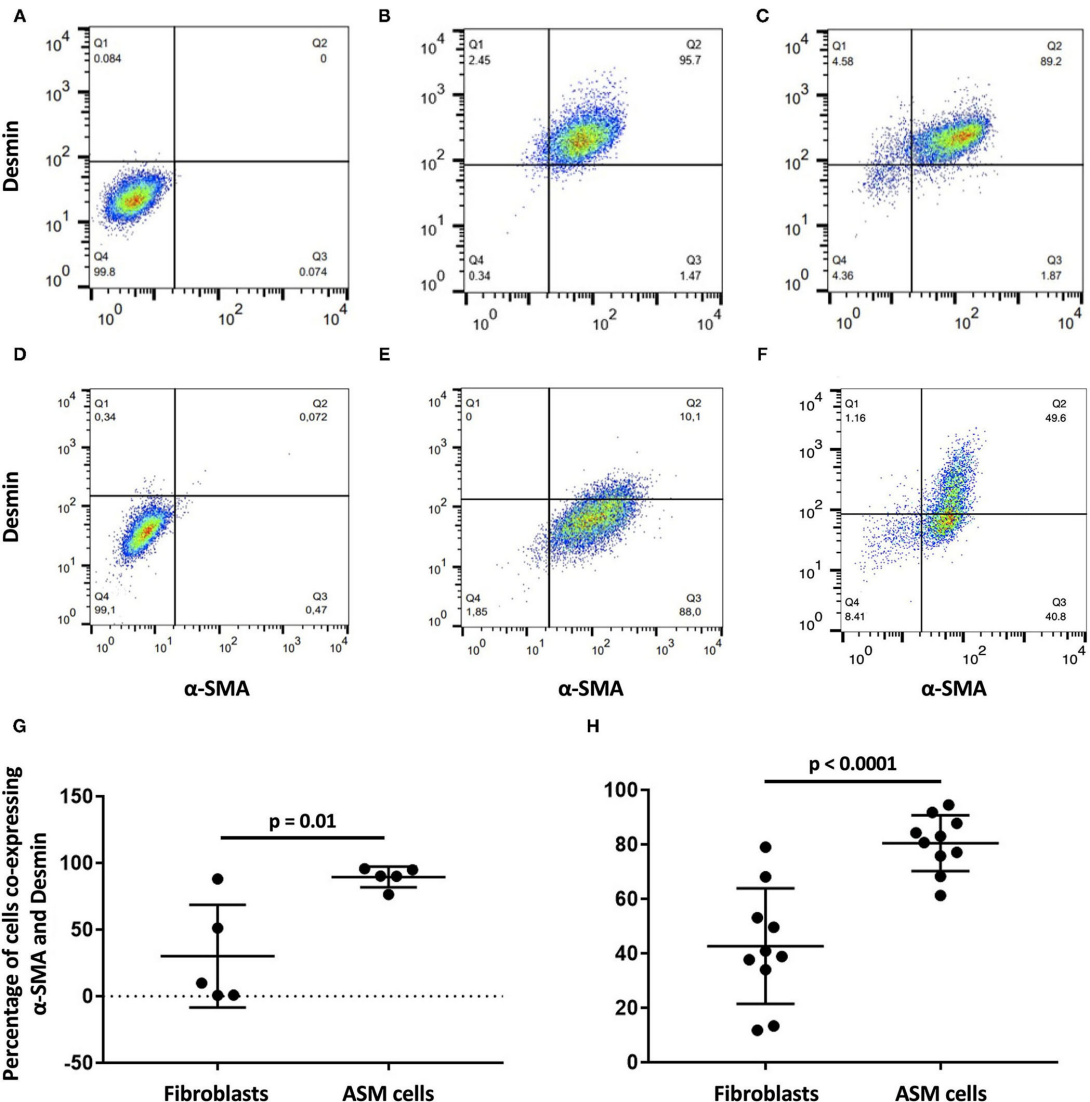
α-SMA and desmin co-expression in airway smooth muscle (ASM) cells and fibroblasts by flow cytometry. Isotype control quadrant for α-SMA and desmin co-expression, respectively, for **(A)** ASM cells and **(D)** fibroblasts **(D)**. **(G)** Percentage of α-SMA and desmin co-expression is significantly higher in **(B)** ASM cells than **(E)** fibroblasts before recellularization. **(H)** Significance is maintained after recellularization [**(C)** ASM cells and **(F)** fibroblasts].

The protein expression of ASM cells and fibroblasts in the 2D culture and after 31 days of culture with different biological matrices was compared by flow cytometry. As expected for these cell types, the co-expression of desmin and α-SMA was greater in the ASM cells than in the fibroblasts both before (*p* = 0.01) and during recellularization (*p* = 0.0001) (Figure 1). Also, the phenotype of ASM cells remained stable as assessed by the means of fluorescence and percentage of positive cells to α-SMA and desmin. These results confirmed the maintenance of high purity of ASM cells during the 3D recellularization process.

### HEPS Staining and Histomorphometry

**Figure 2:**
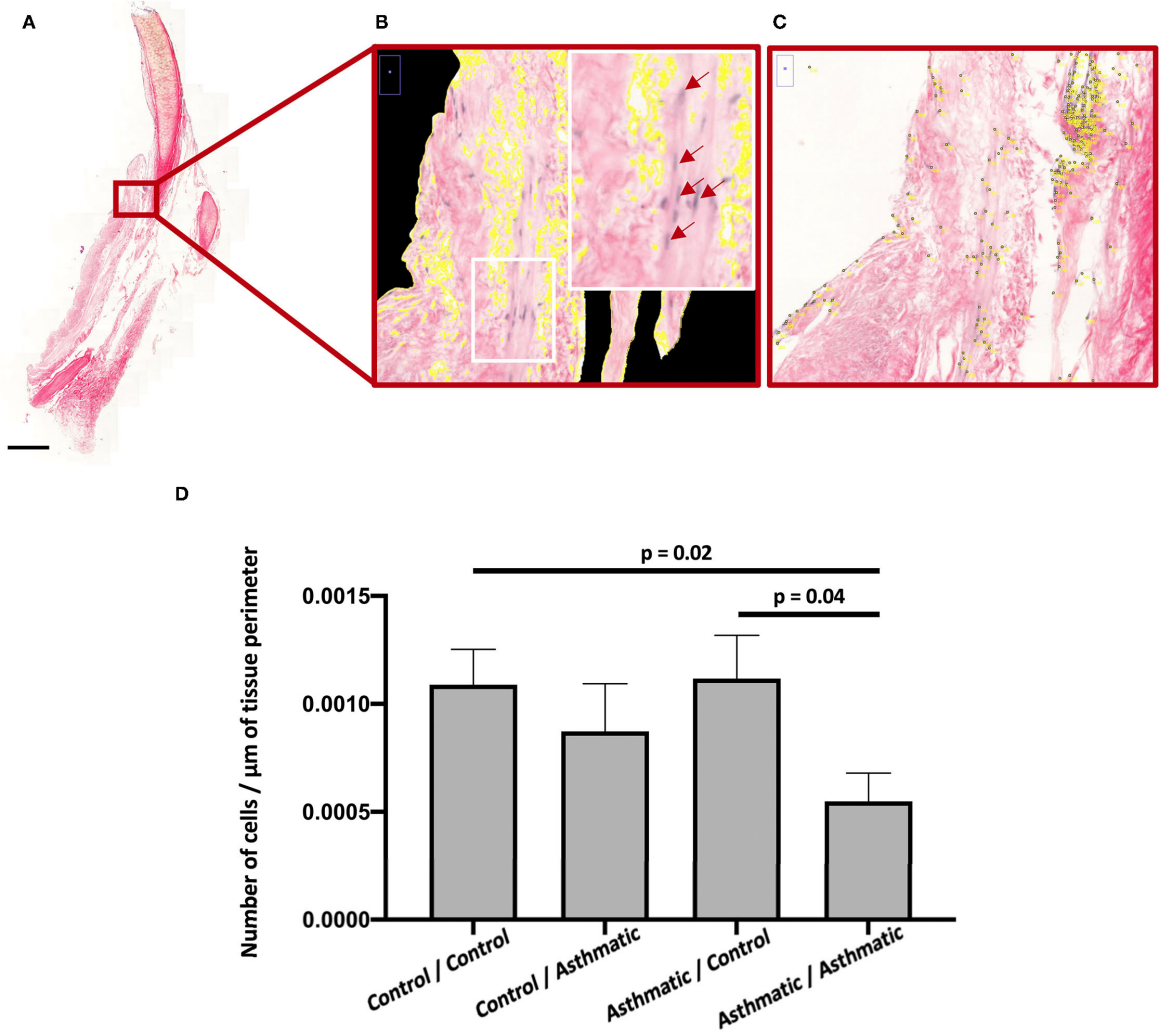
ASM cell proliferation. **(A)** Hematoxylin-eosin-phloxine-safran (HEPS) staining of recellularized matrices at 200 magnification for histomorphometry. **(B)** Zoom on perimeter calculation on a scan section at magnification 200, the white rectangle is focused on a section containing ASM cells stained in purple with some positive nuclei identified by red arrows. **(C)** Point counting zoom on a scan section at magnification 200. **(D)** Number of nuclei per μm in the asthmatic matrix/asthmatic cell group was significantly decreased compared with the control matrix/control cell and asthmatic matrix/asthmatic cell groups.

Histomorphometry revealed an increased number of ASM cells in the control matrices/control cells (*p* = 0.02) and in the asthmatic matrices/control cells (*p* = 0.04) when compared with the asthmatic matrices/asthmatic cells (Figure 2).

### Scan Electron Microscopy of Recellularized Matrix

**Figure 3:**
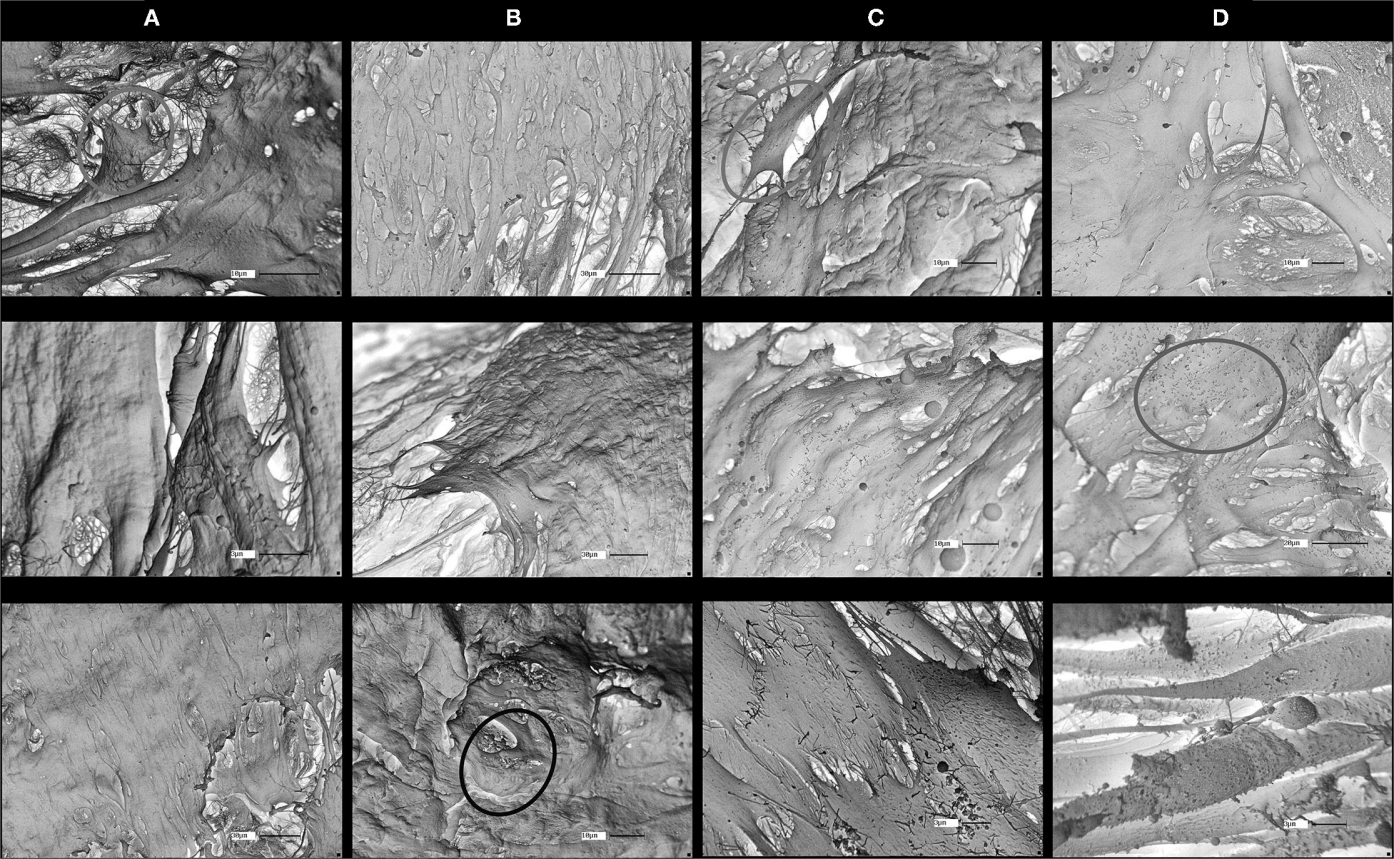
Scan electron microscopy (SEM) of matrices at 41 days of recellularization for the four groups. ASM cells are more evenly distributed and more uniform in **(A)** control matrices/control ASM cells and in **(B)** control matrices/asthmatic ASM cells than in **(C)** asthmatic matrices/control ASM cells and in **(D)** asthmatic matrices/asthmatic ASM cells. Large collagen deposits (yellow circles) are visible in the control matrix/asthmatic ASM cell groups and cell debris (green circle) are present particularly in asthmatic matrices/asthmatic ASM cells. Red circles refer to examples of fibroblast shape. Magnifications are specified on each picture.

Ultrastructural analysis by scan electron microscopy (SEM) of the recellularized scaffolds revealed four distinct groups depending on the health status of horses providing the matrices and ASM cells. On the ECM from healthy horses, ASM cells expressed focal adhesions to the scaffolds and tended to cover the ECM, forming a uniform cell mat. The latter is found but to a lesser extent, when asthmatic ASM cells on matrices from healthy horses are cultured. However, these asthmatic cells expressed larger collagen deposits and small projections on their surface, revealing a continued recellularization process around the fibers. On matrices from asthmatic horses, the recellularization process is unevenly distributed. On these matrices, the ASM cells from healthy horses presented a fibroblastic aspect, and cell debris and senescent cells were observed. These latter findings appeared enhanced when cells from asthmatic horses were used (Figure 3).

**Figure 4:**
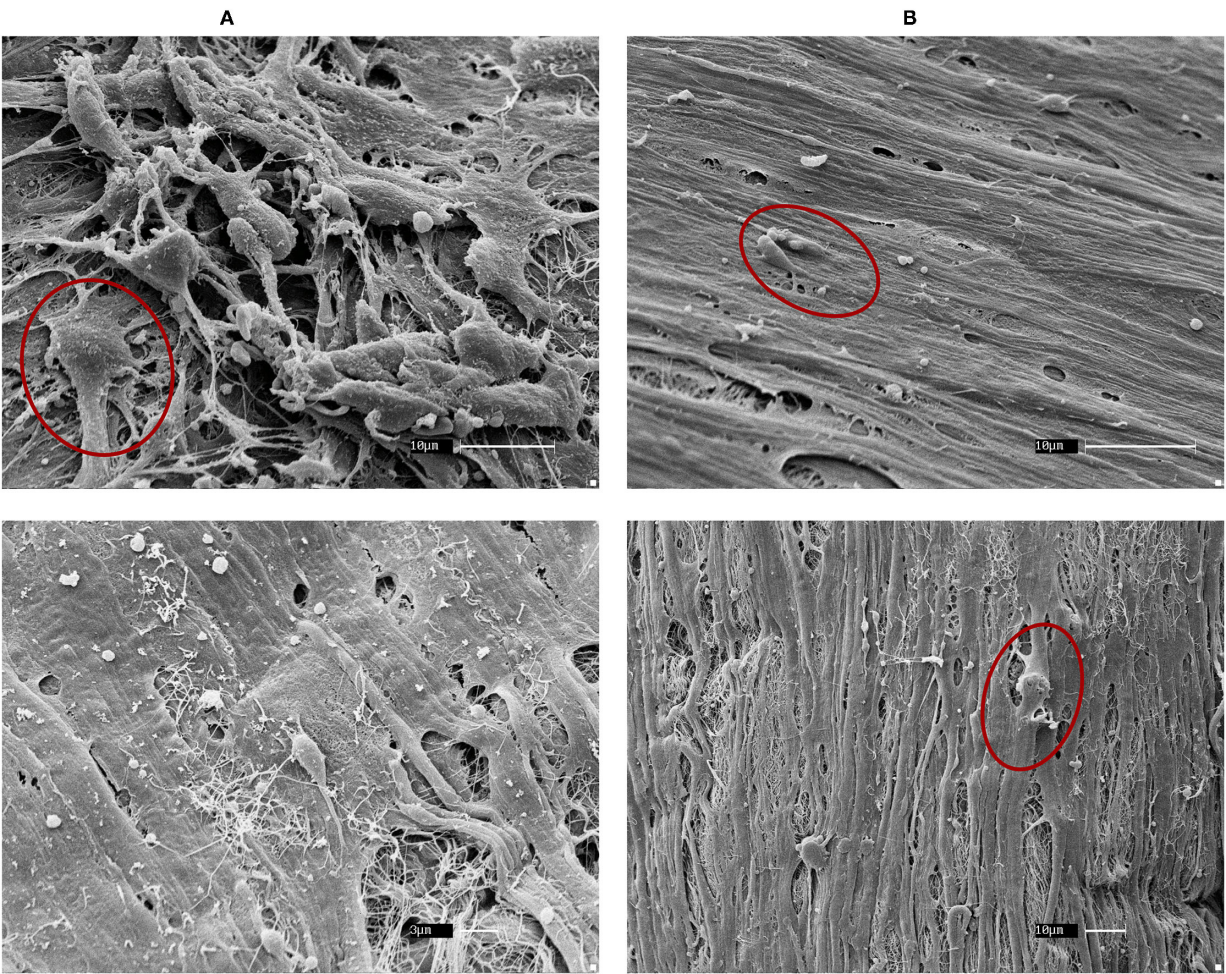
SEM of horse embryonic fibroblast recellularization. Fibroblasts had a good coverage of the matrices and an even distribution in both **(A)** control and **(B)** asthmatic matrices. Red circles show example of fibroblasts. Magnifications are specified on each picture.

All fibroblasts (skin, bronchi, embryos) cultured on healthy and asthmatic matrices demonstrated a good adhesion to all matrices and a different and more homogenous aspect than what was observed with ASM cells (Figure 4).

### Targeted Next Generation Resequencing for Recellularized Bronchi

Six libraries with abnormal gene read distributions at sequencing were excluded. Therefore, statistical analysis were performed to compare control matrices/control cells and asthmatic matrices/asthmatic cells groups with each of the 2 remaining libraries.

The heatmap revealed a cluster of remodeling genes (*HLAB15, HLADQB1, FGA, FGF13, HLA_B8, LYZ, IGHM, IGKC, HNRPDL_HNRNPE, HLA-DMA, HLA-DMB, MLCK*, and *HLA_EQCA-DBQ3*) expressed by the native bronchi but not by the recellularized matrices, suggesting that these genes are not expressed by ASM cells.

**Figure 5:**
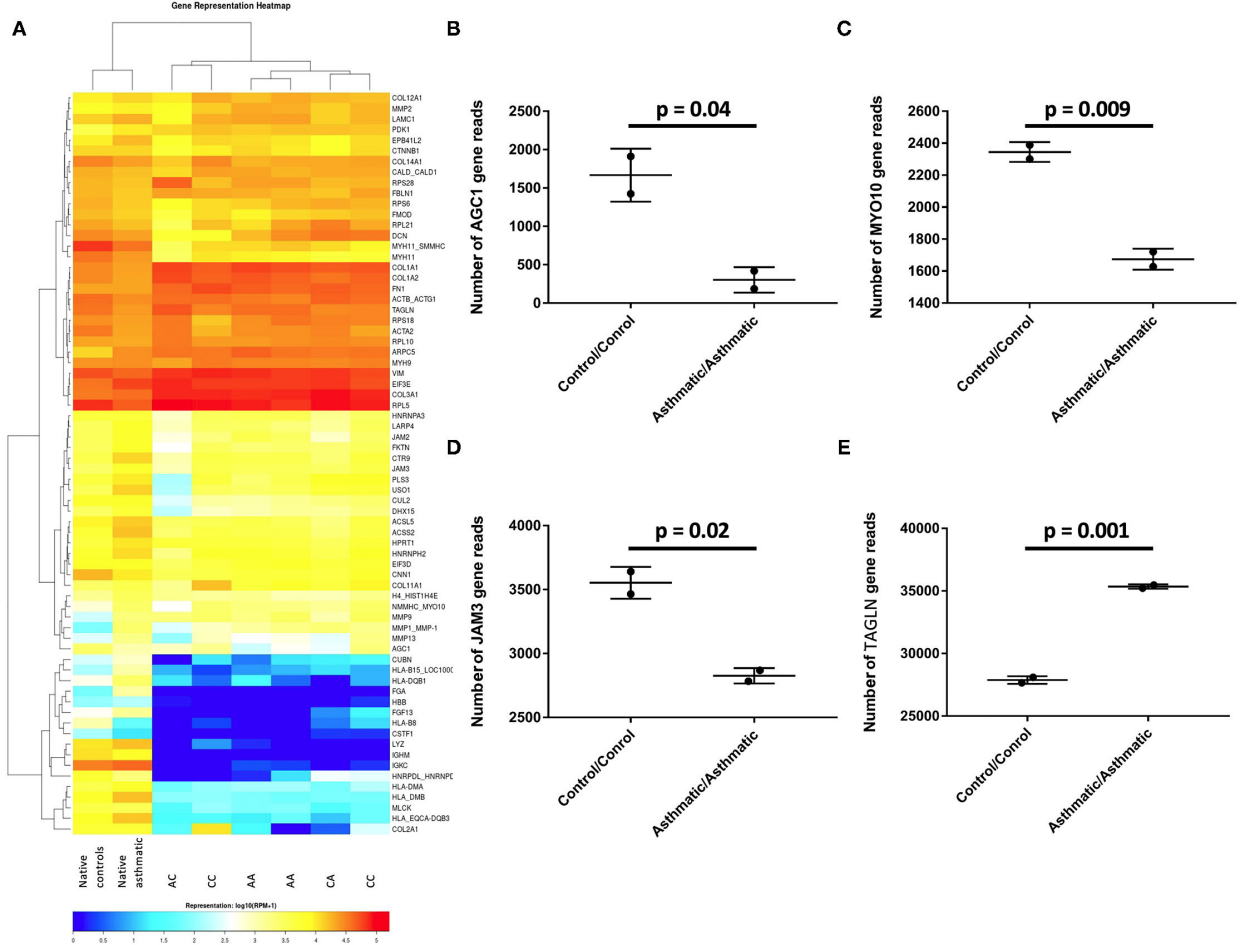
Differentially expressed genes in the control cell/control matrix group vs. the asthmatic cell/asthmatic matrix group. **(A)** Heatmap of all the analyzed groups with CC representing the control matrix/control cell group; CA is the control matrix/asthmatic cell group; AC is the asthmatic matrix/control cell group; AA is the asthmatic matrix/ asthmatic cell group. **(B)** *AGC1*, **(C)** *MYO10*, and **(D)** *JAM3* were significantly upregulated in the control/control group, while **(E)** *TAGLN* was overexpressed in the asthmatic /asthmatic group.

From the 70 genes evaluated, *AGC1* (*p* = 0.04), *MYO10* (*p* = 0.009), and *JAM3* (*p* = 0.02) were upregulated in the control matrices/control cells compared with the asthmatic matrices/asthmatic cells, while *TAGLN* was overexpressed in the asthmatic matrices/asthmatic cells, with *p* = 0.001 (Figure 5).

## Discussion

The ECM is a complex network composed of more than 300 different multi-domain proteins that releases bioactive fragments upon proteolysis (33). Through its composition and architecture, it governs the fundamental behaviors of cells and their characteristics, such as adhesion, migration, differentiation, apoptosis, and contractility (34, 35). It has been assessed that cells cultured on 3D matrices had a different behavior which is closerto *in vivo* conditions than on a 2D culture plates (36, 37). Thus, 3D culture has been widely used in different research studies to study the response of cells to drugs and in diseases (38, 39). Thus, we hypothesized that ASM cells cultured on bronchial scaffolds from asthmatic and healthy horses would display a distinct behavior. Our results confirmed this hypothesis, as we observed that ASM cell senescence process and gene expression varied depending on the health status of the horses from which the matrices originated. Also, there was a reduction in ASM cell number when the cells were harvested from asthmatic airways and cultured on asthmatic matrices, when compared with the control ASM cultured on control matrices. These findings indicate that both the asthmatic ECM and ASM cells may contribute to ASM remodeling in asthma.

The histomorphometry results revealed that ASM cells from healthy horses proliferated more than ASM cells from asthmatic horses whether the matrices were from asthmatic or healthy horses, suggesting that the main effector was health status of the cells. However, SEM and qualitative observations suggested that ASM cell behavior was conditioned both by the health condition of the horse providing the homing matrix and by the health status of the horse providing the cells. However, our finding of reduced number of ASM cells in asthma is in contrast with studies reporting ASM cell proliferation in both human asthma (8, 9) and equine asthma (30, 40). In horses, a dynamic equilibrium associated with increased myocyte proliferation and apoptosis was thought to be limiting ASM mass, preventing the occlusion of the airway lumen (30, 40). Both the markedly reduced cell number on histomorphometry and the presence of senescent cells on SEM we observed when recellularizing scaffolds with ASM cells from asthmatic horses suggest a shift in dynamic equilibrium that is in favor of cell death rather than cell proliferation. Asthmatic bronchial ECMs are stiffer and present altered laminin, elastin, vitronectin, collagen and fibronectin deposition and composition when compared with healthy ECMs (41–45). Considering cell-matrix “inside-out” and “outside-in” cross-talk and interdependence (23, 33), these alterations may result in modifications in the conformation of matrix integrins (23, 46), that in turn, may explain the lack of ASM cell proliferation (11, 47, 48). It is important to point out that previous studies have determined that the decellularization process we used does not alter protein composition, including that of collagen and fibronectin, nor the general architecture of the bronchi (27, 49–51).

The observation of large protein deposits on matrices from healthy horses seeded with cells from asthmatic horses and small projections on the surface of the cells was suggestive of a continued recellularization process. Indeed, it has been shown that vascular smooth muscle cells were hyperplasic on stiffened collagen fibrils (52). Increased cell deaths and senescence when ASM cells from healthy and asthmatic horses are cultured on matrices from asthmatic horses also indicate a contribution of the asthmatic ECM on ASM cell behavior.

The ECM from equine bronchi decellularized using the method implemented in this study was shown to maintain the protein composition of the native bronchi, such as that of fibronectin and collagens I and IV (27), known to promote ASM cell survival and proliferation (53). However, the lack of cell proliferation in the asthma group could be explained by the absence of structural and immune cell types in our study model, as they contribute to asthma remodeling. In equine asthma, neutrophils are the predominant granulocytes present in the airway lumen. It has been reported that ASM cell proliferation increased dose-dependently when cells are exposed to neutrophil elastase (54) or to exosomes from lipopolysaccharide-stimulated-neutrophils (26). On the other hand, neutrophil elastase along with other proteases induced ASM cell apoptosis probably resulting from ECM degradation, as fibronectin degradation products were found in the supernatant of ASM cells exposed to neutrophil elastase (55). This may lead to a dynamic equilibrium limiting ASM proliferation and ECM deposition in asthma.

Ampliseq is a sequencing technique that enables the simultaneous analysis of hundreds of genes with ultra-high multiplexed PCR. The number of reads of each gene provided by the analysis reflects its degree of expression. It is a suitable technic for exploratory studies, as it allows the identification of differentially expressed genes that could be specifically targeted in subsequent analysis. Only 4 of 70 genes related to asthmatic remodeling were differentially expressed between the control matrix/control ASM cell and asthmatic matrix/asthmatic ASM cell groups. *AGC1* encodes for aggrecan, a major component of the cartilaginous extracellular matrix (56) also expressed by vascular smooth muscle cells when cultured *in vitro* on calcified elastin or hydroxyapatite (57). Most of the literature has linked this gene to skeleton development and disease (56, 58, 59), but it has also been associated with atopic asthma (60). *MYO10* is an actin-associated molecular motor known to serve in intracellular movement (61). The *in vivo* role of *MYO10* remains unclear, but *in vitro* it promotes cellular filopodia formation, and cell migration has been reported (62, 63). Therefore, *MYO10* may be promoting ASM cell migration at a higher rate on matrices from healthy horses when compared with those of asthmatic horses. *JAM3* is encoding for junction adhesion molecule C, a protein that regulates different cellular processes (64) and is expressed by smooth muscle cells among other cells (65–68). Using a wild-type murine model of IL-33-induced airway inflammation, downregulation of the expression of *JAM3* and 60 other tight junction-coding genes was observed (69). Similarly, *JAM3* expression was downregulated in the asthmatic/asthmatic group compared with control/control one, further supporting a role in asthma. *TAGLN* is coding for transglin-2, an actin binding protein that causes its gelation. It is highly expressed in smooth muscles (70), and its expression is upregulated in asthmatic bronchial biopsies in mild asthmatics (71). In agreement with this finding, *TAGLN* was also overexpressed in ASM from asthmatic horses cultured on asthmatic bronchial matrices when compared with ASM and ECM from healthy animals. Of note, other genes may have been found differently expressed with larger sample sizes.

Flow cytometry results, comparing between ASM cells and fibroblasts, confirmed the cell purity over the recellularization process, as the high co-expression of α-SMA and desmin is only displayed by the ASM cells (72, 73). Furthermore, desmin is expressed by mature muscle cells but not by fibroblasts (73). This ensured that the results obtained in this study reflect the interaction outcomes between ASM cells and bronchial extracellular matrices, as our model did not consider ASM cell interaction with other cell types.

To the best of our knowledge, this is the first attempt to evaluate the effects of asthmatic ECM on ASM cell phenotype using a biological bronchial matrix. Our results support that bronchial ECM health status determines the gene expression and behaviors of ASM cells, such as adhesion, migration, and, possibly, survival. Asthmatic ASM cells are more receptive to the influence of ECM from asthmatic origins, suggesting that the health status of both cells and matrices is the determinant of the outcome of ECM/ASM interactions in asthma. However, the model we used had limitations, namely, it did not consider the interactions of ASM cells with other structural and inflammatory cell types. Further studies including more cell types (epithelial cells, neutrophils, fibroblasts) in this 3D model would be of interest to assess the effect of ASM cell interactions with other cells implicated in the pathogenesis of asthma.

## Data Availability Statement

The original contributions presented in the study are publicly available. This data can be found here: https://doi.org/10.5683/SP3/4V2OBE.

## Ethics Statement

The animal study was reviewed and approved by Ethical Committee of the University of Montreal.

## Author Contributions

SB participated in the design of the study, isolated ASM cells, sampled and decellularized the bronchi, cultured the ASM cells on the decellularized bronchi, sampled the 3D constructs for SEM, performed the histologic staining, conducted MOVAT and HePS staining and histomorphometry, and collected and analyzed the results of this study. MM and GS prepared and analyzed the scan microscopy images. GB did the statistical analysis of histomorphometry results and approved the statistical analysis of Amliseq and flowcytometry. J-PL participated in the design of the study, analysis of the results, and supervised the experimental protocols. All authors contributed to the article and approved the submitted version.

## Funding

The Canadian Institutes of Health Research, Canada (CIHR) (Grant No: PJT-148807) and the faculty of superior and postdoctoral studies (FESP) scholarships funded this study. The funders had no role in study design, data collection and analysis, decision to publish, or preparation of the manuscript.

## Conflict of Interest

The authors declare that the research was conducted in the absence of any commercial or financial relationships that could be construed as a potential conflict of interest.

## Publisher's Note

All claims expressed in this article are solely those of the authors and do not necessarily represent those of their affiliated organizations, or those of the publisher, the editors and the reviewers. Any product that may be evaluated in this article, or claim that may be made by its manufacturer, is not guaranteed or endorsed by the publisher.

## Supplementary Material

Supplementary Material 1 Schematic representation of study design. Blue arrows: airway smooth muscle (ASM) cells; green arrows: fibroblasts; blue arrows on thick red arrows: ASM cells recellularizing bronchial matrices; green on thick red arrows: fibroblasts recellularizing bronchial matrices.

Supplementary Material 2 Ante-mortem information of the six horses. P_L_, maximum variation in transpulmonary pressure during a breath; E_L_, pulmonary elastance; R_L_, pulmonary resistance. BALF, broncho-alveolar lavage cytology, 400 cells were evaluated for differential counts (%).

Supplementary Material 3 Panel of the 70 customized equine-specific probes for genes used for Ampliseq library construction and sequencing.

## Abbreviations

ASM: airway smooth muscle
ECM: extracellular matrix
SEM: scan electron microscopy.
