# Supplementary figures and images for: Asthmatic Bronchial Matrices Determine the Gene Expression and Behavior of Smooth Muscle Cells in a 3D Culture Model
Source: Front Allergy. 2021 Nov 26;2:762026. doi: 10.3389/falgy.2021.762026 (PMC8974673; doi:10.3389/falgy.2021.762026)

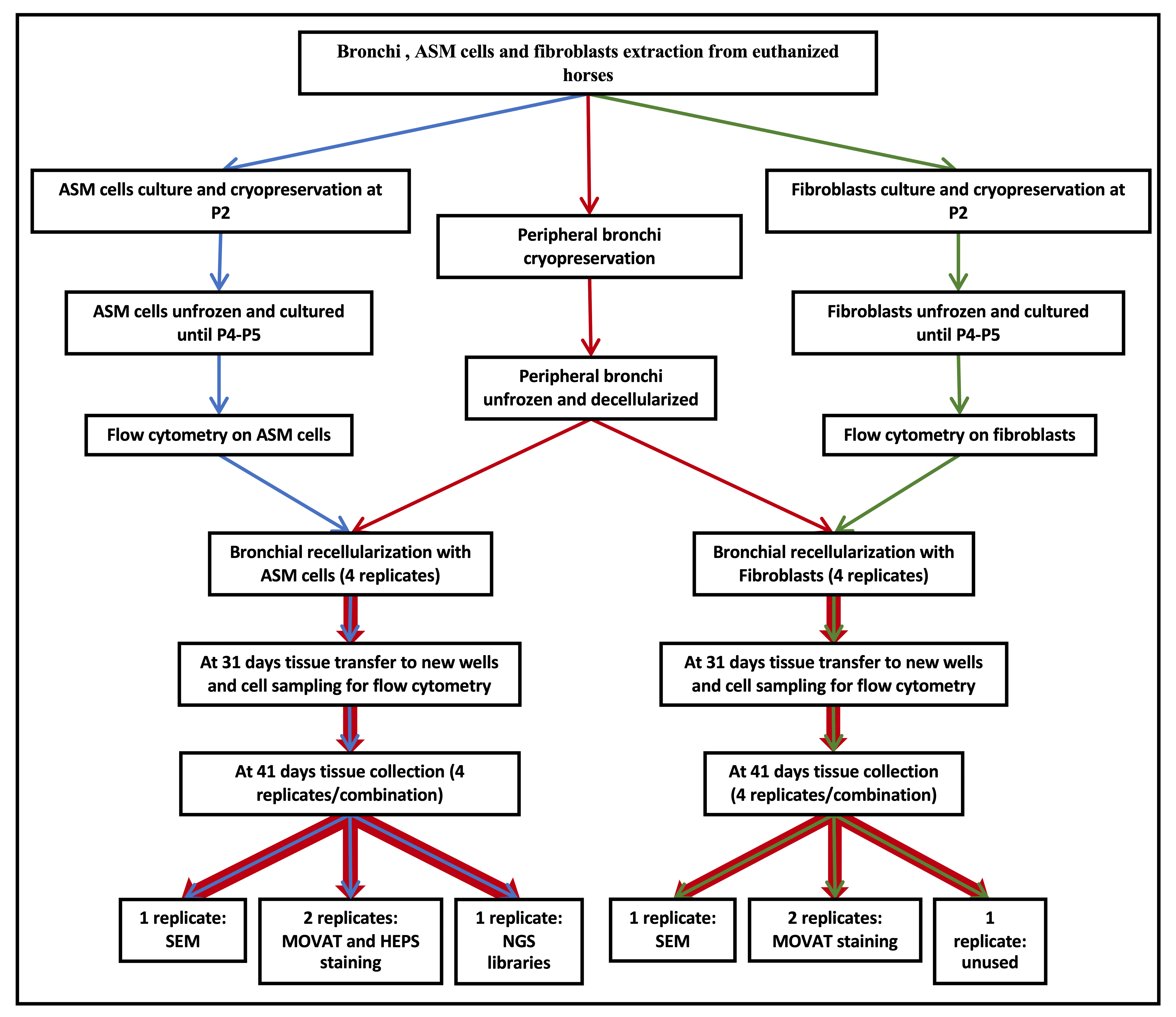

Supplement: Supplementary Material 1 — Schematic representation of study design. Blue arrows: airway smooth muscle (ASM) cells; green arrows: fibroblasts; blue arrows on thick red arrows: ASM cells recellularizing bronchial matrices; green on thick red arrows: fibroblasts recellularizing bronchial matrices. [file Image_1.TIFF]
