# Supplementary material for: Asthmatic Bronchial Matrices Determine the Gene Expression and Behavior of Smooth Muscle Cells in a 3D Culture Model
Source: Front Allergy. 2021 Nov 26;2:762026. doi: 10.3389/falgy.2021.762026 (PMC8974673; doi:10.3389/falgy.2021.762026)
Supplement: Supplementary Material 2 — Ante-mortem information of the six horses. PL, maximum variation in transpulmonary pressure during a breath; EL, pulmonary elastance; RL, pulmonary resistance. BALF, broncho-alveolar lavage cytology, 400 cells were evaluated for differential counts (%). [file Table_1.DOCX]

|  | **Control horses** | | | **Asthmatic horses** | | |
| --- | --- | --- | --- | --- | --- | --- |
| **Sex** | Mare | Mare | Mare | Mare | Gelding | Mare |
| **Age (years)** | 9 | 10 | 9 | 16 | 15 | 19 |
| **E_L_ (cm H_2_O/L)** | 0.78 | 0.43 | 0.37 | 12.58 | 1.5 | 7.56 |
| R_L_ **(cm H_2_O/L/s)** | 0.61 | 0.44 | 0.37 | 3.64 | 1.99 | 4.10 |
| **BALF cell counting** |  | | | | | |
| **Neutrophils** | 3.75% | 9.75% | 8.4% | 34.75% | 81.5% | 16% |
| **Eosinophils** | 0% | 0% | 1% | 0% | 0% | 0% |
| **Mastocytes** | 0.25% | 0% | 1.5% | 0.25% | 0.5% | 0.75% |
| **Basophiles** | 0% | 0% | 0% | 0% | 0% | 0% |
| **Lymphocytes** | 46% | 22.27% | 49.8% | 42.5% | 9% | 54.5% |
| **Macrophages** | 50% | 68% | 38.4% | 22.5% | 9% | 29% |
